# PSYCHOSOCIAL RESEARCHER PERSPECTIVES OF THE SUMMER RESEARCH INSTITUTE AND TRAINING NEEDS

**DOI:** 10.1093/geroni/igae098.1121

**Published:** 2024-12-31

**Authors:** Alyssa Lanzi

**Affiliations:** University of Delaware, Newark, Delaware, United States

## Abstract

Over 30 public psychosocial early career researchers have participated in the Alzheimer’s Association Interdisciplinary Summer Research Institute (AA-ISRI) across the first three years of the program. During the week spent at AA-ISRI, psychosocial participants attend a variety of sessions, including both full group sessions and track-specific sessions; receive mentoring from peers and expert faculty; and refine their research proposals. Psychosocial track participants gain exposure to a wide range of potential intervention research populations and designs and enhance skills in methods for ADRD research including identifying and developing measures, utilizing mixed methods, and how to initiate and follow through with community-engaged research. The ultimate hope of the psychosocial track of the AA-ISRI is that participants will depart having formed long-term connections and the tools needed to advance their research careers. During this presentation, AA-ISRI psychosocial alum Dr. Alyssa Lanzi will describe the experience of AA-ISRI as a participant, including the highlights and helpfulness of the didactic sessions offered at the program, the mentoring and networking opportunities that were available, and the impact that AA-ISRI had on the short-term career trajectory and research. For example, since AA-ISRI, Dr. Lanzi has received an R01 funded by NIA/NIH to establish an open-access database of connected speech/language samples from older adults at-risk for dementia to study language as a potential biomarker. A key feature of this proposal is its community engagement plan to enroll those from minoritized backgrounds. This includes working with collaborators from across the country including two researchers Dr. Lanzi met at AA-ISRI.

